# CK2 regulates somatostatin expression in pancreatic delta cells

**DOI:** 10.1080/19382014.2025.2515332

**Published:** 2025-06-05

**Authors:** Selina Wrublewsky, Annika Clemenz, Anne S. Boewe, Cedric Wilden, Caroline Bickelmann, Claudia Götz, Patrick E. MacDonald, Matthias W. Laschke, Emmanuel Ampofo

**Affiliations:** a Institute for Clinical and Experimental Surgery, Saarland University, Homburg, Germany; b Medical Biochemistry and Molecular Biology, Saarland University, Homburg, Germany; c Department of Pharmacology and Alberta Diabetes Institute, University of Alberta, Edmonton, Alberta, Canada

**Keywords:** Pancreatic islets, pancreatic δ-cells, PDX1, protein kinase CK2, somatostatin

## Abstract

Pancreatic and duodenal homeobox protein (PDX)1 is a major transcription factor for the regulation of insulin, glucagon and somatostatin (SST) expression. PDX1 is phosphorylated by CK2 and inhibition of this kinase results in an increased insulin and decreased glucagon secretion. Therefore, we speculated in this study that CK2 also affects SST expression. To test this, we analyzed the effects of the two CK2 inhibitors CX-4945 and SGC as well as of PDX1 overexpression on SST expression and secretion in RIN14B cells by qRT-PCR, luciferase assays, Western blot and ELISA. SST expression and secretion were additionally assessed in isolated murine and human islets exposed to the CK2 inhibitors. Moreover, we determined the expression and secretion of the pancreatic endocrine hormones in CX-4945-treated mice. We found a suppressed SST expression in RIN14B cells due to a methylated SST promoter, which could be abolished by DNA demethylation. Under these conditions, we showed that CK2 inhibition increases SST gene expression and secretion. Additional experiments with overexpression of a CK2-phosphorylation mutant of PDX1 verified that SST expression is regulated by CK2. The exposure of isolated murine and human islets to CX-4945 or SGC as well as the treatment of mice with CX-4945 revealed that CK2 also regulates SST expression under physiological conditions. Taken together, these findings not only demonstrate that CK2 controls SST expression in pancreatic δ-cells but also emphasize the crucial role of this kinase in regulating the main hormones of the endocrine pancreas.

## GRAPHICAL ABSTRACT

**Figure:**
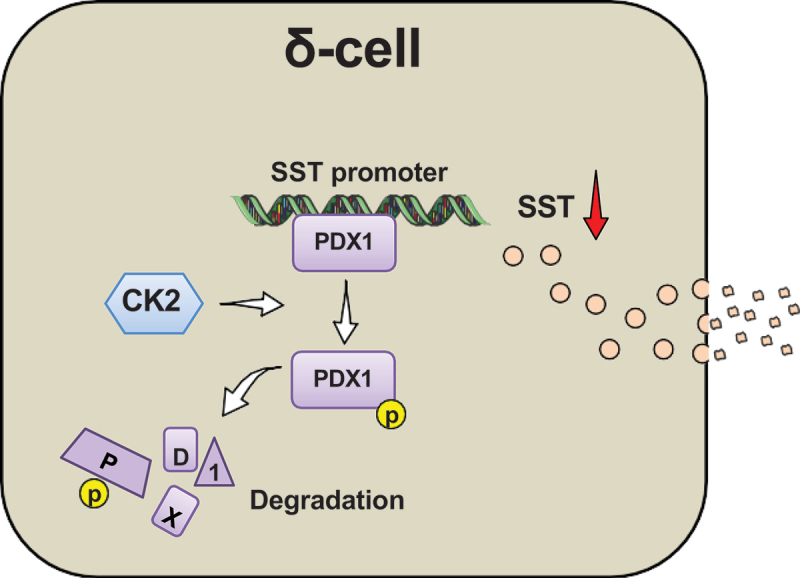
CK2 phosphorylates PDX1 in δ-cells, which promotes its degradation. This, in turn, reduces SST expression and, thus, secretion.

## Introduction

Protein kinase CK2, a highly conserved serine/threonine kinase, is ubiquitously expressed in eukaryotes. CK2 consists of two catalytic α- or α′-subunits and two non-catalytic β-subunits and is known to phosphorylate more than 500 different substrates. Accordingly, CK2 is involved in the regulation of several metabolic processes.^1–5^ In this context, the kinase determines the activity of enzymes, such as glycogen synthase and glucose-6-phosphate isomerase.^6,7^ Moreover, it has been demonstrated that CK2 controls endocrine pancreatic functions by downregulating the activity of the transcription factor pancreatic and duodenal homeobox protein (PDX)1.^8,9^

In pancreatic β-cells, PDX1 induces the expression of the insulin gene and other genes responsible for glucose sensing and metabolism, such as glucose transporter (GLUT)2 and glucokinase.^10,11^ In contrast, PDX1 has a repressive function on glucagon expression in β-cells.^12^ Hence, it is not surprising that pancreatic α-cells, which physiologically highly express glucagon, exhibit low levels of PDX1.^13^ We have already demonstrated that CK2 regulates the expression of insulin and glucagon in pancreatic islets via phosphorylation of PDX1. Further analyses showed that the loss of the CK2-dependent phosphorylation of PDX1 on threonine 231 and serine 232 stabilizes and increases the transcriptional activity.^8,14,15^ This results in an upregulated insulin and downregulated glucagon expression.^8,9,16^ Besides the expression of PDX1 in α- and β-cells, this transcription factor is also expressed in δ-cells. In these cells, PDX1 is capable of stimulating the expression of the peptide hormone somatostatin (SST) via binding to specific consensus sites on the SST promoter.^17,18^

SST is produced by the proteolytic cleavage of pro-SST into the two cyclic peptides SST-28 and SST-14 amino acids.^19^ The latter one is the major final product in pancreatic δ-cells.^20^ SST is stored in secretory granules and its secretion is regulated by dietary components, such as amino acids, glucose and fat.^21–23^ The hormone has a short half-life (~2 min) in the circulation and binds to specific somatostatin receptors (SSTR) on various tissues, which reduces cell proliferation, cell migration, angiogenesis and hormone release.^24–28^ Of note, an elevated expression of SSTR can be found on pathological tissues, such as neuroendocrine tumors, and SST analogues are commonly used for the treatment of these types of cancer.^29–31^

Since (i) CK2 inhibition promotes PDX1 activity, (ii) PDX1 stimulates the expression of SST and (iii) δ-cells express CK2 and PDX1, we hypothesized that CK2 inhibition increases SST expression in δ-cells. To test this, we exposed the δ-cell line RIN14B and murine islets to the two CK2 inhibitors CX-4945 and SGC and determined SST expression. Moreover, mice were treated with CX-4945 to study the systemic effect of CK2 inhibition on endocrine pancreatic hormone expression. Finally, we analyzed the inhibitory effect of CK2 inhibition on SST expression and release of human islets.

## Results

### CK2 inhibition does not affect SST expression in RIN14B cells

**Figure 1.**
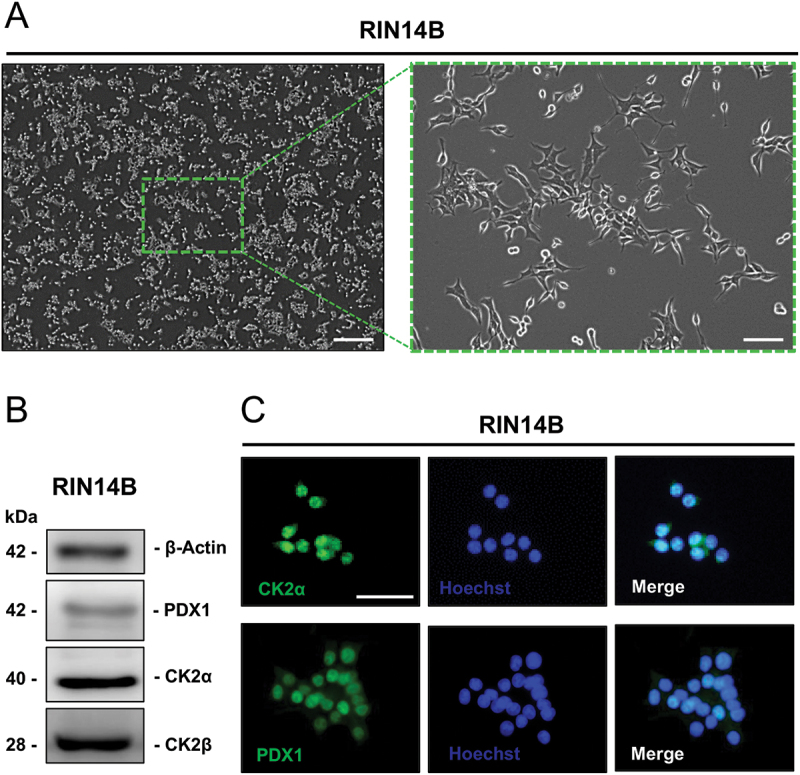
Expression of CK2 in RIN14B cells. (A) bright field images showing the morphology of RIN14B cells (scale bar in left panel: 800 µm; scale bar in right panel: 200 µm). (B) Representative Western blots of β-actin, PDX1, CK2α, CK2β expression in whole cell extracts of RIN14B cells. (C) Representative immunofluorescence stainings of CK2α (green, upper panel) and PDX1 (green, lower panel) in RIN14B cells. Cell nuclei were stained with Hoechst 33,342 (blue). Scale bar: 50 µm.

To study the effect of CK2 inhibition on SST expression, we used the RIN14B cell line (Figure 1A). We first assessed the expression of CK2α, CK2β and PDX1. We found that the proteins are expressed in these cells as shown by Western blot analyses (Figure 1B). Additional immunofluorescence stainings of CK2 and PDX1 revealed that both proteins are mainly localized in the nucleus (Figure 1C).

**Figure 2.**
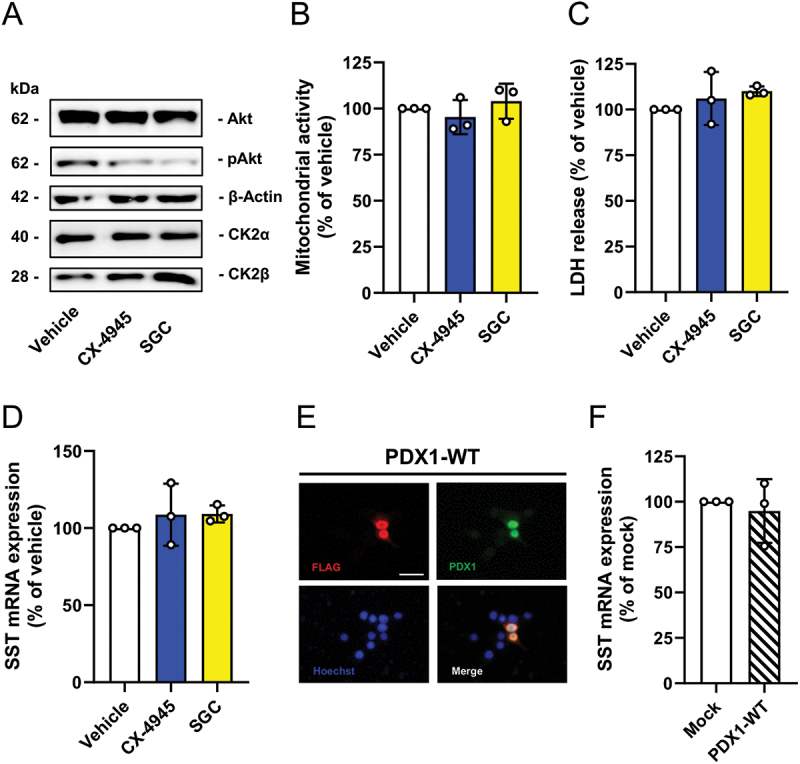
CK2 inhibition does not affect viability and SST expression in RIN14B cells. (A) Representative Western blots of Akt, pAkt, β-actin, CK2α and CK2β expression in whole cell extracts of RIN14B cells exposed to CX-4945, SGC or vehicle for 24 h. (B and C) RIN14B cells were treated as described in (A) and the viability was analyzed by a WST-1 assay (B) and LDH assay (C). Data are expressed in % of vehicle (*n* = 3 each). Mean ± SD. (D) Quantitative analysis of SST mRNA expression in RIN14B cells treated as described in (A). Data are expressed in % of vehicle (*n* = 3 each). Mean ± SD. (E) Representative immunofluorescence stainings of RIN14B cells overexpressing FLAG-tagged PDX1-WT. FLAG-Tag (red, upper left panel) and PDX1 (green, upper right panel) in RIN14B cells. Cell nuclei were stained with Hoechst 33,342 (blue). Scale bar: 25 µm. (F) Quantitative analysis of SST mRNA expression in RIN14B cells overexpressing FLAG-tagged PDX1-WT or control vector (mock). Data are expressed in % of mock (*n* = 3 each). Mean ± SD.

To reduce CK2 activity, we performed a pharmacological strategy. CK2 inhibition is mainly caused by targeting the ATP-binding site, which may result in off-target effects due to the highly conserved nature of the ATP pocket.^32,33^ To exclude that the herein observed effects are caused by off-target effects, we used two specific CK2 inhibitors CX-4945^34^ and SGC.^33^ Moreover, the cells were exposed to only 10 µM of each inhibitor to reduce their inhibitory effect on further kinases.^16,35,36^ Our results demonstrate that the two inhibitors markedly reduce the phosphorylation of Akt on serine 129 in RIN14B cells (Figure 2A), a specific CK2 phosphorylation site,^37^ without affecting cell viability, as shown by water-soluble tetrazolium (WST) and lactate dehydrogenase (LDH) assays (Figure 2(B,C)). Surprisingly, we detected no effect of CK2 inhibition on SST gene expression (Figure 2D). To trigger SST gene expression in RIN14B cells, we overexpressed PDX1-wildtype (WT) in this cell line (Figure 2E). However, again we did not observe an increased SST gene expression (Figure 2F).

### SST promoter methylation represses SST expression in RIN14B cells

**Figure 3.**
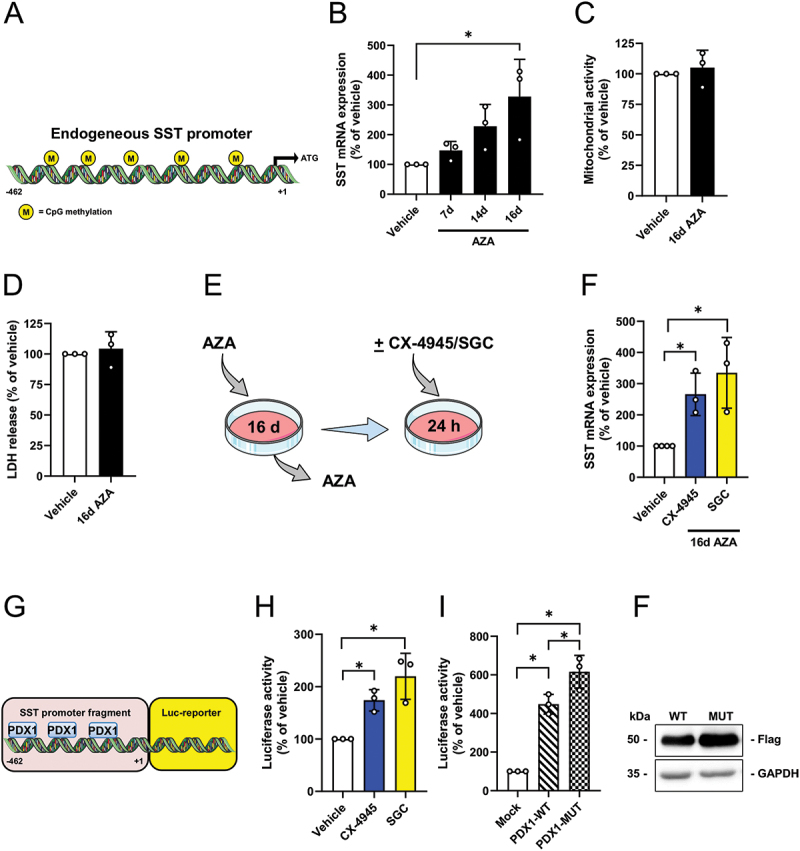
SST promoter hypermethylation represses SST expression in RIN14B cells. (A) Schematic illustration of the methylated endogenous SST promoter region. (B) RIN14B cells were exposed to AZA for the indicated time points and SST mRNA expression was assessed. Data are expressed in % of vehicle (*n* = 3 each). Mean ± SD. *p < 0.05. (C and D) RIN14B cells were exposed to AZA for 16 d and their viability was analyzed by a WST-1 assay (C) and LDH assay (D). Data are expressed in % of vehicle (*n* = 3 each). Mean ± SD. (E) Schematic illustration of the cell treatment strategy. RIN14B cells were exposed to AZA for 16 d and subsequently treated with CX-4549, SGC or vehicle. (F) RIN14B cells were treated as described in (E) and SST mRNA expression was assessed. Data are expressed in % of vehicle (*n* = 3 each). Mean ± SD. *p < 0.05. (G) Schematic illustration of the PDX1 binding sites of the SST promoter reporter gene construct. (H) RIN14B cells were transfected with pGL4-SST for 24 h and subsequently exposed to CX-4945, SGC or vehicle for 24 h. The cells were lysed and the luciferase activity was detected by a luciferase assay. Data are expressed in % of vehicle (*n* = 3 each). Mean ± SD. *p < 0.05. (I) RIN14B cells were transfected with pGL4-SST and FLAG-tagged PDX1-WT or FLAG-tagged PDX1-MUT for 24 h. Cells transfected with pGL4-SST and control vector served as control (mock). The cells were lysed and the luciferase activity was detected by a luciferase assay. Data are expressed in % of mock (*n* = 3 each). Mean ± SD. *p < 0.05. (**F**) Representative Western blot of RIN14B cells, which were transfected with FLAG-tagged PDX1-WT or FLAG-tagged PDX1-MUT for 24 h. PDX1 was detected by FLAG antibodies. GAPDH acts as loading control.

It has been reported that hypermethylation of the SST promoter may decrease SST gene expression^38^ (Figure 3A). Based on our results showing that CK2 inhibition as well as PDX1 overexpression do not affect SST gene expression, we next treated RIN14B cells with the DNA methyltransferase (DNMT) inhibitor 5-aza-2’-deoxycytidine (AZA) to demethylate the SST promoter region. Our results clearly showed that the exposure of the cells to AZA increases SST gene expression over a time period of 16 days (Figure 3B). Of note, 16 days of AZA-treatment did not affect cell viability (Figure 3(C,D)). We then pre-treated the cells with AZA for 16 days and subsequently exposed them for 24 h to CX-4945, SGC and vehicle (Figure 3E). As expected, this treatment resulted in elevated levels of SST mRNA in cells exposed to the two CK2 inhibitors when compared to vehicle-treated controls (Figure 3F). We additionally performed SST-promoter analyses. For this purpose, we cloned the SST promoter fragment harboring several PDX1 binding sites into a luciferase reporter vector (Figure 3G) and subsequently transfected RIN14B cells with this construct. We found a higher luciferase activity in cells exposed to the two CK2 inhibitors (Figure 3H). To verify that this effect is mediated by a higher transcriptional activity of PDX1 due to the reduced CK2 activity, we overexpressed PDX1-WT and PDX1-mutant (MUT). The latter cannot be phosphorylated by CK2. We showed that PDX1-WT and PDX1-MUT increased luciferase activity (Figure 3I). Of note, luciferase activity in PDX1-MUT-overexpressing cells was superior to that of PDX1-WT-overexpressing cells (Figure 3I). As expected, additional Western blot analyses demonstrated that loss of the CK2-dependent phosphorylation of PDX1 (PDX1-Mut) leads to its stabilization (Figure 3J). This indicates that CK2 activity may regulate SST expression via PDX1 phosphorylation.

### CK2 inhibition increases SST expression and secretion in murine isolated islets

**Figure 4.**
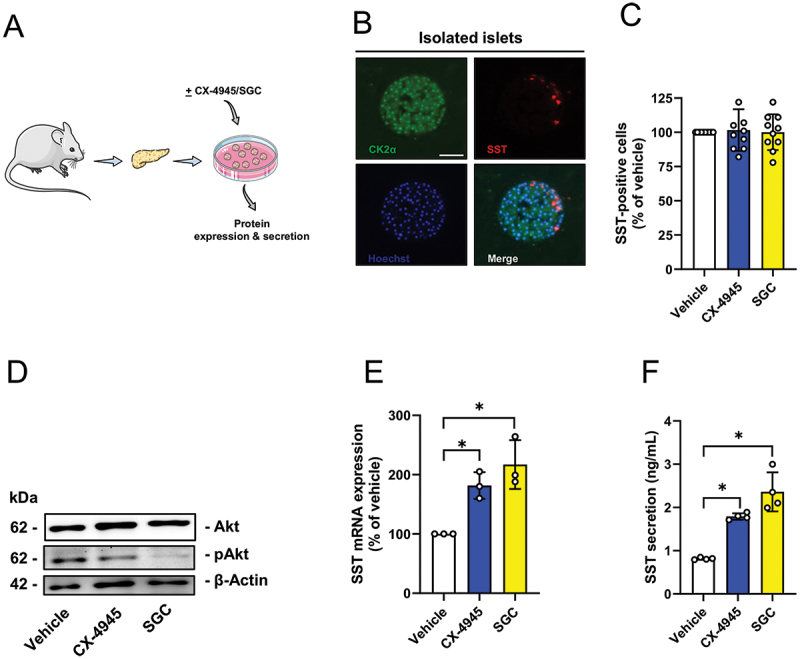
CK2 inhibition increases SST expression and secretion in murine isolated islets. (A) Schematic illustration of the experimental setting: islets were isolated from mice and exposed to CX-4945, SGC or vehicle for 24 h. Subsequently, the islets were collected and prepared for protein expression and secretion analyses. (B) Representative immunofluorescence stainings of CK2α (green) and SST (red) in isolated murine islets. Cell nuclei were stained with Hoechst 33,342 (blue). Scale bar: 100 µm. (C) Isolated murine islets were treated as described in (A) and the number of SST-positive cells was determined in % of vehicle-treated islets. Mean ± SD. (D) Representative Western blots of Akt, pAkt and β-actin in whole cell extracts of murine islets treated as described in (A). (E) Murine islets were treated as described in (A) and SST mRNA expression was assessed. Data are expressed in % of vehicle (*n* = 3 each). Mean ± SD. *p < 0.05. (F) Murine islets were treated as described in (A) and SST secretion was quantitatively analyzed (ng/mL) (*n* = 3 each). Mean ± SD. *p < 0.05.

To study the effect of CK2 inhibition on SST expression ex vivo, we isolated islets from murine pancreata and exposed them to CX-4945, SGC or vehicle (Figure 4A). Thereafter, we analyzed the effect of CK2 inhibition on SST expression. Immunofluorescence stainings showed that somatostatin-expressing δ-cells are positive for CK2 (Figure 4B). To exclude that the exposure of isolated islets to the two inhibitors affects their cellular composition, we first determined the number of δ-cells in CX-4945- and SGC-exposed islets. Our results showed no differences between the groups (Figure 4C). Next, we assessed the effect of CK2 inhibition on SST expression. We found that both inhibitors efficiently diminished the phosphorylation of Akt on serine 129 resulting in a significantly upregulated SST mRNA expression (Figure 4(D,E)). In addition, we also detected higher levels of secreted SST in CX-4945- and SGC-exposed islets when compared to vehicle-treated controls (Figure 4F).

### CK2 inhibition increases SST expression under physiological conditions

**Figure 5.**
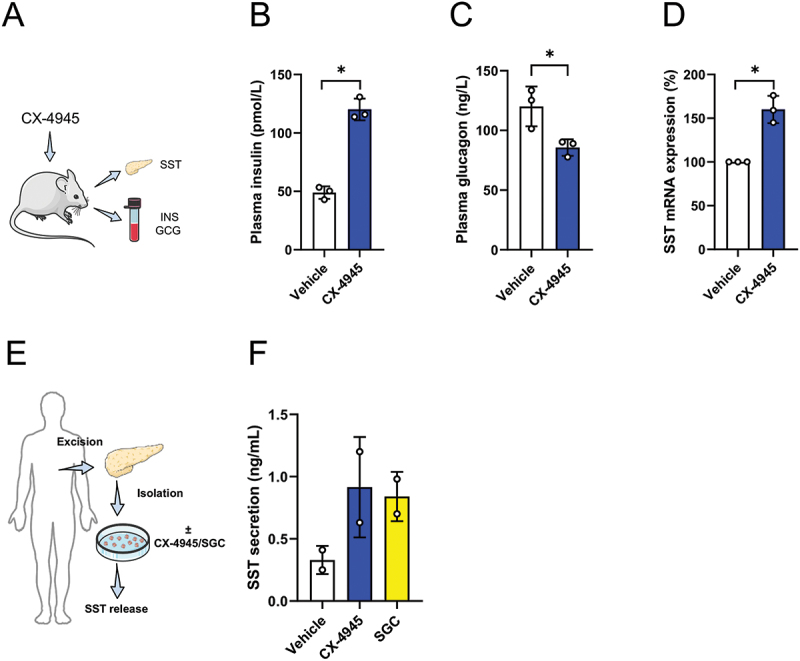
CK2 inhibition increases SST expression under physiological conditions. (A) Schematic illustration of the experimental setting: mice were treated with CX-4945 or vehicle. After 3 d, the mice were euthanized and islets as well as plasma were isolated to study SST gene expression, insulin and glucagon plasma levels. (B and C) Quantitative analysis of plasma insulin levels (pmol/L) and plasma glucagon levels (ng/L) of mice treated as described in (A) (*n* = 3 each). Mean ± SD. *p < 0.05. (D) SST mRNA expression of isolated islets from mice treated as described in (A). Data are expressed in % of vehicle (*n* = 3 each). Mean ± SD. *p < 0.05. (E) Schematic illustration of the experimental setting: islets were isolated from pancreata of human donors. The islets were then exposed to CX-4945, SGC or vehicle and the subsequent SST secretion was assessed. (F) Human islets were treated as described in (E) and SST secretion was quantitatively analyzed (ng/mL) (*n* = 2 each). Mean ± SD.

To assess the effect of the increased SST expression after CK2 inhibition under physiological conditions, mice were treated with CX-4945 or vehicle for 3 days and plasma insulin/glucagon as well as tissue SST expression were examined (Figure 5A). We found elevated plasma insulin levels and lower glucagon levels in mice after CK2 inhibition (Figure 5(B,C)). Moreover, we detected a higher SST expression in mice treated with CX-4945 when compared to controls (Figure 5D). We finally determined SST secretion after CK2 inhibition in isolated islets from healthy human donors (Figure 5E). Our results clearly showed that CX-4945 and SGC also significantly increase SST secretion in human islets (Figure 5F).

## Discussion

In previous studies, we could already demonstrate that protein kinase CK2 plays an important role in the regulation of endocrine pancreatic hormones.^2^ In this context, we have shown that CK2 inhibition promotes insulin expression and secretion.^9^ In addition, we detected a decreased glucagon secretion after reducing CK2 activity.^16^ Molecular analyses revealed that both effects are mainly caused by the increased transcriptional activity of PDX1 after CK2 inhibition.^8,16^ In the present study, we now found that CK2 inhibition increases SST expression and secretion of pancreatic δ-cells in a PDX1-dependent manner indicating the important role of CK2 in the glucometabolic control.

We first analyzed the effect of CK2 inhibition on SST expression in the cell line RIN14B. This cell line is a secondary clone derived from the RIN-m rat islet cell line, which has been shown to synthesize and secrete SST.^39^ In the present study, we found that RIN14B expresses CK2 and PDX1. In contrast, we detected low mRNA levels of SST, which were not affected by the treatment with CX-4945 or SGC and PDX1 overexpression. In addition, we did not measure SST in the supernatant of high glucose-stimulated RIN14B cells. This is in line with the results from Branstrom et al.^40^ showing that RIN14B cells stimulated for 30 min with 25 mm K^+^ or 500 μM tolbutamide do not release SST. Hence, it is conceivable that this δ-cell line undergoes dedifferentiation and loses its capability of secreting SST or that SST gene expression is repressed by DNA modification.

SST is a protein with anti-proliferative and anti-secretory activity.^41,42^ Therefore, the specific suppression of SST gene expression is a prerequisite for proper cell proliferation. Epigenetic alterations, such as DNA cytosine methylation of CpG sites in oncogenes and tumor-suppressor genes, have been extensively described in cancer cells and various cell lines.^43,44^ This process is mainly catalyzed by DNMT. Of note, the SST gene is hypermethylated in different stages of tumors^45–47^ and demethylation drugs can restore SST gene expression.^48,49^ Hence, we assume that this epigenetic machinery also silences SST gene expression in the herein used RIN14B cells. In fact, we could show that the DNMT inhibitor AZA promotes the expression of SST. In addition, CK2 affects SST expression, as shown by a higher expression of SST after CK2 inhibition. We have reported that CK2 phosphorylates PDX1 and this phosphorylation represses its transactivation activity by reducing PDX1 stability.^2^ It is known that the SST promoter harbors several PDX1 consensus sites,^50^ which are important for SST gene expression.^17^ Accordingly, the elevated SST expression after CX-4945 or SGC exposure may be caused by increased PDX1 stability and, thus, activity. Indeed, we found that overexpression of the phosphorylation-deficient mutant of PDX1 increases SST expression when compared to overexpression of WT PDX1.

We next used murine and human isolated pancreatic islets to evaluate the effect of CK2 inhibition on SST expression. In line with our in vitro results, we found that CK2 inhibition significantly reduces the expression and secretion of SST. This indicates that the CK2-dependent regulation of SST expression is species-independent. However, it should be noted that the complex paracrine communication between α-, β-, δ-cells may additionally affect SST expression. For instance, Svendsen et al.^51^ showed that exogenous glucagon stimulates SST secretion through glucagon and glucagon like peptide (GLP)-1 receptors. We reported that CK2 inhibition reduces glucagon secretion.^16^ Hence, it is conceivable that the herein observed increased SST expression is weakened by the decreased glucagon secretion after CK2 inhibition.

In this study, we could show that the treatment of mice with CX-4945 leads to higher plasma insulin levels and lower plasma glucagon levels. This is in line with our previous study showing that systemic CK2 inhibition affects the secretion of the two hormones.^16^ We were not able to measure SST plasma levels after CK2 inhibition. This is most probably due to the extremely short half-life (~2 min) of SST.^28,52^ To overcome this problem, we determined SST expression in pancreatic tissue of CX-4945-treated mice. As expected, we detected an upregulated SST expression.

Besides pancreatic islets, SST is expressed in additional organs, such as the central nervous system, stomach and intestine.^47,53^ SST predominantly exerts endocrine and exocrine inhibitory effects across multiple systems, such as cell proliferation, cell migration, hormone secretion and angiogenesis.^53,54^ Hence, synthetic analogues of SST are widely used in clinical practice for the treatment of neuroendocrine tumors and acromegaly, which are associated with a high SSTR expression.^30,31,35,53,55^ The overexpression of CK2 in cancer promotes cell proliferation and migration by dysregulating signaling pathways, such as nuclear factor-kappa (NF-κ)B and phosphoinositide 3-kinase (PI3K)/Akt.^56^ Based on these central functions of CK2 in tumorigenesis, it is not surprising that a broad spectrum of CK2 inhibitors has been developed, culminating in the synthesis of CX-4945.^57^ This compound has a high bioavailability^57^ and is currently tested in phase I and II clinical trials for the treatment of different cancer types, such as medulloblastoma (NCT03904862) and multiple myeloma (NCT01199718). Therefore, it is conceivable that targeting CK2 by CX-4945 represents a promising therapeutic approach for the treatment of SSTR-positive tumors not only by upregulation of SST expression but also by repressing further oncogenic pathways.

In the present study, we found that CK2 inhibition increased SST expression in δ-cells of murine and human islets. The analysis of the underlying mechanisms revealed that this was due to an elevated transcriptional activity of PDX1. These results together with the results of previous studies showing that CK2 inhibition increases insulin and decreases glucagon expression, clearly illustrate the crucial role of CK2 in regulating glucose homeostasis.

## Materials and methods

### Materials

Collagenase NB 4 G was purchased from SERVA Electrophoresis GmbH (Heidelberg, Germany). Neutral red solution, Tween 20 and Hoechst 33,342 were purchased from Sigma-Aldrich (Taufkirchen, Germany). Ketamine (Ursotamin®) was purchased from Serumwerk Bernburg (Bernburg, Germany) and Xylazine (Rompun®) from Bayer (Leverkusen, Germany). Bovine serum albumin (BSA) and fetal calf serum (FCS) were purchased from Santa Cruz Biotechnology (Heidelberg, Germany). Cell lysis reagent QIAzol and QuantiNova Reverse Transcription Kit were purchased from Qiagen (Hilden, Germany). HepatoQuick® as well as WST and LDH assays were purchased from Roche (Basel, Switzerland). The qScriber cDNA Synthesis Kit and ORA SEE qPCR Green ROX L Mix were purchased from HighQu (Kraichtal, Germany). The CK2 inhibitors CX-4945 and SGC (SGC-CK2-1) were purchased from SelleckChem (Munich, Germany). Protein assay dye reagent and luminol-enhanced chemiluminescence (ECL) Western blotting substrate were purchased from Bio-Rad Laboratories (Feldkirchen, Germany). Lipofectamine 3000, Roswell Park Memorial Institute (RPMI) medium 1640 and insulin ELISA kit were purchased from Fisher Scientific GmbH (Schwerte, Germany). Somatostatin ELISA kit was purchased from Phoenix Pharmaceuticals (Burlingame, USA). Glucagon ELISA kit was purchased from R&D systems (Minneapolis, USA).

## Antibodies

The anti-pAktS129 antibody (ab133458) was from Abcam (Cambridge, UK). The anti-AKT antibody (11E7) was from Cell Signaling (Frankfurt am Main, Germany). The anti-CK2β antibody (E9) was from Santa Cruz Biotechnology (Heidelberg, Germany). The anti-β-Actin (AC-74) and the anti-FLAG were (F1804) were from Sigma Aldrich (Taufkirchen, Germany). The generation of the anti-CK2α antibody was described previously.^58^ PDX1 was identified with a polyclonal antiserum generated by immunizing rabbits with recombinant mouse PDX1.^14^ The peroxidase-labeled anti-rabbit antibody (NIF 824) and the peroxidase-labeled anti-mouse antibody (NIF 825) were purchased from GE Healthcare (Freiburg, Germany).

## Cell culture

The rat RIN-14B cells (ATCC: CRL-2059) were cultivated in RPMI supplemented with 10% (v/v) FCS in a humidified atmosphere with 5% CO_2_ at 37°C. Transient transfection was performed with the SST promoter construct or different p3×FlagCMV7.1-PDX1-constructs^59^ and Lipofectamine 3000 according to the manufacturer’s protocol. The used cell line was free from mycoplasma contamination.

## Western blot analysis

RIN-14B cells or isolated murine islets exposed to CX-4945 (10 µM), SGC (10 µM) or DMSO as control for 24 h were harvested and lysed for 30 min at 4°C with lysis buffer (10 mmol/L Tris-HCl, pH 7.5, 10 mmol/L NaCl, 0.1 mmol/L EDTA, 0.5% (v/v) Triton X-100, 0.02% NaN_3_ (w/v) supplemented with 0.5 mmol/L phenylmethylsulfonyl fluoride (PMSF) and a protease and phosphatase inhibitor cocktail (1:75 v/v, Sigma-Aldrich)). The cytoplasmic and nuclear extracts were generated and analyzed, as described previously in detail.^60^

## qRT-PCR

Total RNA from RIN-14B cells as well as isolated murine islets exposed to CX-4945, SGC or DMSO for 24 h were extracted using QIAzol lysis reagent. The corresponding cDNA was synthesized from the total RNA by QuantiNova Reverse Transcription Kit and the qRT-PCR analysis was performed by means of ORA^TM^ SEE qPCR Green ROX L Mix (highQu, Kraichtal, Germany). Primer sequences for qRT-PCR were coded as follows: mouse SST forward 5′-CCCAACCAGACAGAGAATGA −3′ and reverse 5′- ACAGGATGTGAATGTCTTCCA −3′; rat SST forward 5′- GGAAGACATTCACATCCTG −3′ and reverse 5′- GCAGGGTCTAGTTGAGCAT-3′; GAPDH forward 5′-CGGTGCTGAGTATGTC-3′ and reverse 5′-TTTGGCTCCACCCTTC-3′.

## WST-1 assay

A WST-1 assay was used to analyze the effect of CK2 inhibition on cell viability by determining the activity of mitochondrial dehydrogenases. Cells were seeded in a 96-well culture plate at a density of 2 × 10^3^ cells/well. After 24 h, a WST assay was performed according to the manufacturer’s protocol.

## LDH assay

A LDH assay was used to evaluate the cytotoxic effects of CK2 inhibition. Cells were seeded in a 96-well culture plate at a density of 2 × 10^3^ cells/well. After 24 h, a LDH assay was performed according to the manufacturer’s protocol.

## Reporter luciferase assay

The sequence of the mouse SST promoter was amplified and the resulting construct was cloned into the XhoI restriction site of the luciferase reporter vector pGL4.10 (Promega, Mannheim, Germany). The identity of pGL4.10-glucagon was verified by sequencing. The transcriptional activity of the SST promoter was assessed by reporter gene assays according to the manufacturer’s instructions (Promega, Mannheim, Germany). Briefly, RIN-14B cells were seeded in a 24-well plate. Subsequently, cells were transfected with pGL4 (Mock) or pGL4-SST reporter vector by using Lipofectamine 3000 for 24 h. In addition, pGL4-SST-transfected cells were exposed to CX-4945, SGC or DMSO for 24 h. Then, cells were lysed and the luciferase activity was detected by a luminescence plate reader.

## Immunofluorescence microscopy

RIN14B cells were seeded on coverslips, fixed with PBS (3.7 % formalin) for 10 min and subsequently permeabilized with PBS (0.2 % Triton X-100) for 30 min. Afterward, the cells were blocked in PBS (2 % BSA) for further 30 min at room temperature. The cells were then incubated with specific primary antibodies (1:50), which were detected by the corresponding fluorescence-coupled secondary antibodies (1:250). Subsequently, the cells were sealed with mounting media and analyzed by fluorescence microscopy (BX60; Olympus, Hamburg, Germany).

## Animals

All animal care and experimental procedures were performed according to the German legislation on protection of animals and the National Institutes of Health (NIH) Guide for the Care and Use of Laboratory Animals (Institute of Laboratory Animal Resources, National Research Council, Washington DC, USA). The experiments were approved by the local governmental animal protection committee (Landesamt für Verbraucherschutz LAV Saarland). Animals were maintained on a standard 12/12 h day/night cycle. Standard pellet chow (Altromin, Lage, Germany) and water were provided ad libitum.

## Isolation of murine and human pancreatic islets

Murine pancreatic islets were isolated by collagenase-induced enzymatic digestion and purified by hand picking, as described previously in detail.^61^ Isolated islets were cultivated in RPMI 1640 supplemented with 10% (v/v) FCS, 100 U/mL penicillin and 0.1 mg/mL streptomycin for 24 h at 37°C and 5% CO_2_. For the determination of SST secretion, we used 30 islets per 24-well.

Human islets were isolated from donor pancreata at the Alberta Diabetes Institute IsletCore (http://www.isletcore.ca.)^62^ of the University of Alberta (Edmonton, Alberta, Canada) and were cultured in low‐glucose (5.5 mmol/L) DMEM with L‐glutamine, 110 mg/L sodium pyruvate, 10% FBS and 100 units/mL penicillin/streptomycin. All human islet studies were approved by the Human Research Ethics Board (Pro00013094; Pro00001754) at the University of Alberta and all families of organ donors provided written informed consent.

## Immunohistochemical analyses

Murine islets were incubated for 45 min at 37°C in 100 µL HepatoQuick®, 50 µL human citrate plasma and 10 µL 10% CaCl_2_ solution. The resulting clot was also fixed for 24 h in 4% paraformaldehyde at 4°C. After dehydration, the paraffin-embedded samples were cut into 3-μm-thick sections. Antigens in samples were demasked by citrate buffer and the unspecific binding sites were blocked by goat serum. Cells were stained with specific primary antibodies (1:300), which were detected by the corresponding fluorescence-coupled secondary antibodies (1:1000). Cell nuclei were stained with Hoechst 33,342. The sections were analyzed using a BX60F fluorescence microscope (Olympus).

## SST secretion measurements

The amount of secreted SST was measured by a SST ELISA kit. For this purpose, RIN14B cells, 30 murine or 20 human islets were cultivated in KRB buffer (140 mm NaCl, 3.6 mm KCl, 2.6 mm CaCl_2_H_2_O, 0.5 mm MgSO_4_7 h_2_O, 0.5 mm NaH_2_PO_4_, 2 mm NaHCO_3_, 5 mm HEPES, 1 mm glucose) for 30 min at 37 °C and 5% CO_2_. Subsequently, the buffer was removed, and the islets were cultivated in KRB buffer (70 mm NaCl, 70 mm KCl, 2.6 mm CaCl_2_H_2_O, 0.5 mm MgSO_4_7 h_2_O, 0.5 mm NaH_2_PO_4_, 2 mm NaHCO_3_, 5 mm HEPES, 20 mm glucose) for 2 h. The supernatants were collected, and the amount of secreted SST was determined by using a SST ELISA kit according to the manufacturer’s protocol.

## Analysis of plasma insulin and glucagon levels in mice

C57BL/6J mice were daily treated by intraperitoneal injection of CX-4945 (1.5 mg/kg dissolved in DMSO/PBS) for 3 days. Mice were killed and the blood was collected. Glucagon secretion was analyzed by a glucagon ELISA kit and insulin by an insulin ELISA kit.

## Statistical analysis

All in vitro and ex vivo experiments were reproduced at least three times. Differences between two groups were assessed by an unpaired Student’s t-test. One way ANOVA was applied when comparing multiple groups. This was followed by the Tukey post-hoc test by means of Prism software 8 (GraphPad). The results were expressed as mean ± SD. Statistical significance was indicated as *p < 0.05.

## References

[1] Al Quobaili F, Montenarh M. CK2 and the regulation of the carbohydrate metabolism. Metabolism. 2012 Nov. 61(11):1512–14. doi: 10.1016/j.metabol.2012.07.011. 22917893

[2] Ampofo E, Nalbach L, Menger MD, Montenarh M, Götz C. Protein kinase CK2-A putative target for the therapy of diabetes mellitus? Int J Mol Sci. 2019 Sep 7. 20(18):4398. doi: 10.3390/ijms20184398. 31500224 PMC6770776

[3] Ampofo E, Schmitt BM, Laschke MW, Menger MD. Function of protein kinase CK2 in thrombus formation. Platelets. 2018 Sep. 11(4):1–7. doi: 10.1080/09537104.2018.1513474. 30204035

[4] Gibson SA, Benveniste EN. Protein kinase CK2: an emerging regulator of immunity. Trends Immunol. 2018 Feb. 39(2):82–85. doi: 10.1016/j.it.2017.12.002. 29307449 PMC5800982

[5] Litchfield DW. Protein kinase CK2: structure, regulation and role in cellular decisions of life and death. Biochemical J. 2003 Jan 1. 369(Pt 1):1–15. doi: 10.1042/bj20021469. PMC1223072 12396231

[6] Hao Y, Ren T, Huang X, Li M, Lee J-H, Chen Q, Liu R, Tang Q. Rapid phosphorylation of glucose-6-phosphate dehydrogenase by casein kinase 2 sustains redox homeostasis under ionizing radiation. Redox Biol. 2023 Sep. 65:102810. doi: 10.1016/j.redox.2023.102810. 37478541 PMC10404535

[7] Queiroz-Claret C, Jolivet P, Chardot T, Bergeron É, Meunier J-C. Time-co-ordinated control of glycogen synthase, protein phosphatase 2A and protein kinase CK2 during culture growth in Yarrowia lipolytica in relation to glycogen metabolism. C R Acad Sci III. 2000 Mar. 323(3):257–266. doi: 10.1016/S0764-4469(00)00127-X. 10782329

[8] Meng R, Al-Quobaili F, Müller I, Götz C, Thiel G, Montenarh M. CK2 phosphorylation of pdx-1 regulates its transcription factor activity. Cell Mol Life Sci. 2010 Jul. 67(14):2481–2489. doi: 10.1007/s00018-010-0348-0. 20339896 PMC11115922

[9] Meng R, Götz C, Montenarh M. The role of protein kinase CK2 in the regulation of the insulin production of pancreatic islets. Biochem Biophys Res Commun. 2010 Oct 15. 401(2):203–206. doi: 10.1016/j.bbrc.2010.09.028. 20849816

[10] Kaneto H, Miyatsuka T, Kawamori D, Yamamoto K, Kato K, Shiraiwa T, Katakami N, Yamasaki Y, Matsuhisa M, Matsuoka T-A, et al. PDX-1 and MafA play a crucial role in pancreatic β-cell differentiation and maintenance of mature β-cell function. Endocr J. 2008 May. 55(2):235–252. doi: 10.1507/endocrj.K07E-041. 17938503

[11] Ohlsson H, Karlsson K, Edlund T. IPF1, a homeodomain-containing transactivator of the insulin gene. EMBO J. 1993 Nov. 12(11):4251–4259. doi: 10.1002/j.1460-2075.1993.tb06109.x. 7901001 PMC413720

[12] Gosmain Y, Cheyssac C, Heddad Masson M, Dibner C, Philippe J. Glucagon gene expression in the endocrine pancreas: the role of the transcription factor Pax6 in α-cell differentiation, glucagon biosynthesis and secretion. Diabetes Obes Metab. 2011 Oct. 13 Suppl 1(s1):31–38. doi: 10.1111/j.1463-1326.2011.01445.x. 21824254

[13] Blodgett DM, Nowosielska A, Afik S, Pechhold S, Cura AJ, Kennedy NJ, Kim S, Kucukural A, Davis RJ, Kent SC, et al. Novel observations from next-generation RNA sequencing of highly purified human adult and fetal islet cell subsets. Diabetes. 2015 Sep. 64(9):3172–3181. doi: 10.2337/db15-0039. 25931473 PMC4542439

[14] Welker S, Gotz C, Servas C, Laschke MW, Menger MD, Montenarh M. Glucose regulates protein kinase CK2 in pancreatic beta-cells and its interaction with PDX-1. Int J Biochem Cell Biol. 2013 Dec. 45(12):2786–2795. doi: 10.1016/j.biocel.2013.10.002. 24126110

[15] Klein S, Meng R, Montenarh M, Götz C. The phosphorylation of PDX-1 by protein kinase CK2 is crucial for its stability. Pharmaceuticals (Basel). 2016 Dec 28. 10(1):2. doi: 10.3390/ph10010002. 28036027 PMC5374406

[16] Ampofo E, Pack M, Wrublewsky S, Boewe AS, Spigelman AF, Koch H, MacDonald PE, Laschke MW, Montenarh M, Götz C, et al. CK2 activity is crucial for proper glucagon expression. Diabetologia. 2024 Jul. 67(7):1368–1385. doi: 10.1007/s00125-024-06128-1. 38503901 PMC11153270

[17] Lu M, Miller C, Habener JF. Functional regions of the homeodomain protein IDX-1 required for transactivation of the rat somatostatin gene. Endocrinology. 1996 Jul. 137(7):2959–2967. doi: 10.1210/endo.137.7.8770920. 8770920

[18] Miller CP, McGehee RE, Habener JF. IDX-1: a new homeodomain transcription factor expressed in rat pancreatic islets and duodenum that transactivates the somatostatin gene. The EMBO J. 1994 Mar 1. 13(5):1145–1156. doi: 10.1002/j.1460-2075.1994.tb06363.x. 7907546 PMC394923

[19] Warren TG, Shields D. Expression of preprosomatostatin in heterologous cells: biosynthesis, posttranslational processing, and secretion of mature somatostatin. Cell. 1984 Dec. 39(3 Pt 2):547–555. doi: 10.1016/0092-8674(84)90461-6. 6150766

[20] Francis BH, Baskin DG, Saunders DR, Ensinck JW. Distribution of somatostatin-14 and somatostatin-28 gastrointestinal-pancreatic cells of rats and humans. Gastroenterology. 1990 Nov. 99(5):1283–1291. doi: 10.1016/0016-5085(90)91151-U. 1976560

[21] DiGruccio MR, Mawla AM, Donaldson CJ, Noguchi GM, Vaughan J, Cowing-Zitron C, van der Meulen T, Huising MO. Comprehensive alpha, beta and delta cell transcriptomes reveal that ghrelin selectively activates delta cells and promotes somatostatin release from pancreatic islets. Mol Metab. 2016 Jul. 5(7):449–458. doi: 10.1016/j.molmet.2016.04.007. 27408771 PMC4921781

[22] Olofsson CS, Salehi A, Gopel SO, Holm C, Rorsman P. Palmitate stimulation of glucagon secretion in mouse pancreatic alpha-cells results from activation of L-type calcium channels and elevation of cytoplasmic calcium. Diabetes. 2004 Nov. 53(11):2836–2843. doi: 10.2337/diabetes.53.11.2836. 15504963

[23] Ipp E, Dobbs RE, Arimura A, Vale W, Harris V, Unger RH. Release of immunoreactive somatostatin from the pancreas in response to glucose, amino acids, pancreozymin-cholecystokinin, and tolbutamide. J Clin Invest. 1977 Sep. 60(3):760–765. doi: 10.1172/JCI108829. 330567 PMC372422

[24] Kossut M, Lukomska A, Dobrzanski G, Liguz-Lęcznar M. Somatostatin receptors in the brain. Postepy Biochemii. 2018 Oct 25. 64(3):213–221. doi: 10.18388/pb.2018_133. 30656906

[25] Klisovic DD, O’Dorisio MS, Katz SE, et al. Somatostatin receptor gene expression in human ocular tissues: RT-PCR and immunohistochemical study. Invest Ophthalmol Vis Sci. 2001 Sep. 42(10):2193–2201. 11527930

[26] Barbieri F, Bajetto A, Pattarozzi A, Gatti M, Würth R, Thellung S, Corsaro A, Villa V, Nizzari M, Florio T, et al. Peptide receptor targeting in cancer: the somatostatin paradigm. Int J Pept. 2013;2013:926295. doi: 10.1155/2013/926295. 23476673 PMC3582104

[27] Theodoropoulou M, Stalla GK. Somatostatin receptors: from signaling to clinical practice. Front Neuroendocrinol. 2013 Aug. 34(3):228–252. doi: 10.1016/j.yfrne.2013.07.005. 23872332

[28] Patel YC, Wheatley T. In vivo and in vitro plasma disappearance and metabolism of somatostatin-28 and somatostatin-14 in the rat. Endocrinology. 1983 Jan. 112(1):220–225. doi: 10.1210/endo-112-1-220. 6128222

[29] Ruscica M, Arvigo M, Steffani L, Ferone D, Magni P. Somatostat in, somatostatin analogs and somatostatin receptor dynamics in the biology of cancer progression. Curr Mol Med. 2013 May. 13(4):555–571. doi: 10.2174/1566524011313040008. 22934849

[30] Godara A, Siddiqui NS, Byrne MM, Saif MW. The safety of lanreotide for neuroendocrine tumor. Expert Opin Drug Saf. 2019 Jan. 18(1):1–10. doi: 10.1080/14740338.2019.1559294. 30582380

[31] Yau H, Kinaan M, Quinn SL, Moraitis AG. Octreotide long-acting repeatable in the treatment of neuroendocrine tumors: patient selection and perspectives. Biologics Targets & Ther. 2017;11:115–122. doi: 10.2147/BTT.S108818. PMC5723116 29255345

[32] Pierre F, Chua PC, O’Brien SE, Siddiqui-Jain A, Bourbon P, Haddach M, Michaux J, Nagasawa J, Schwaebe MK, Stefan E, et al. Discovery and SAR of 5-(3-chlorophenylamino)benzo[c][2,6]naphthyridine-8-carboxylic acid (CX-4945), the first clinical stage inhibitor of protein kinase CK2 for the treatment of cancer. J Med Chem. 2011 Jan 27. 54(2):635–654. doi: 10.1021/jm101251q. 21174434

[33] Wells CI, Drewry DH, Pickett JE, Tjaden A, Krämer A, Müller S, Gyenis L, Menyhart D, Litchfield DW, Knapp S, et al. Development of a potent and selective chemical probe for the pleiotropic kinase CK2. Cell chembiol. 2021;28(546–558):[S2451–9456(20)30522–5 pii; 10.1016/j.chembiol.2020.12.013. PMC8864761 33484635

[34] Siddiqui-Jain A, Drygin D, Streiner N, et al. CX-4945, an orally Bioavailable selective inhibitor of protein kinase CK2, inhibits prosurvival and angiogenic signaling and exhibits antitumor efficacy [70/24/10288 pii].Cancer Res. 2010;70(24):10288–10298. doi: 10.1158/0008-5472.CAN-10-1893. 21159648

[35] Qian ZR, Li T, Ter-Minassian M, Yang J, Chan JA, Brais LK, Masugi Y, Thiaglingam A, Brooks N, Nishihara R, et al. Association between somatostatin receptor expression and clinical outcomes in neuroendocrine tumors. Pancreas. 2016 Nov. 45(10):1386–1393. doi: 10.1097/MPA.0000000000000700. 27622342 PMC5067972

[36] Wells CI, Drewry DH, Pickett JE, Tjaden A, Krämer A, Müller S, Gyenis L, Menyhart D, Litchfield DW, Knapp S, et al. Development of a potent and selective chemical probe for the pleiotropic kinase CK2. Cell Chem Biol. 2021 Apr 15. 28(4):546–558 e10. doi: 10.1016/j.chembiol.2020.12.013. 33484635 PMC8864761

[37] Di Maira G, Salvi M, Arrigoni G, Marin O, Sarno S, Brustolon F, Pinna LA, Ruzzene M. Protein kinase CK2 phosphorylates and upregulates Akt/PKB. Cell Death Differ. 2005 June. 12(6):668–677. doi: 10.1038/sj.cdd.4401604. 15818404

[38] Klomp MJ, Dalm SU, de Jong M, Feelders RA, Hofland J, Hofland LJ. Epigenetic regulation of somatostatin and somatostatin receptors in neuroendocrine tumors and other types of cancer. Rev Endocr Metab Disord. 2021 Sep. 22(3):495–510. doi: 10.1007/s11154-020-09607-z. 33085037 PMC8346415

[39] Gazdar AF, Chick WL, Oie HK, Sims HL, King DL, Weir GC, Lauris V. Continuous, clonal, insulin- and somatostatin-secreting cell lines established from a transplantable rat islet cell tumor. Proc Natl Acad Sci USA. 1980 June. 77(6):3519–3523. doi: 10.1073/pnas.77.6.3519. 6106192 PMC349648

[40] Branstrom R, Hoog A, Wahl MA, Berggren P-O, Larsson O. RIN14B: a pancreatic delta-cell line that maintains functional ATP-dependent K+ channels and capability to secrete insulin under conditions where it no longer secretes somatostatin. FEBS Lett. 1997 Jul 14. 411(2–3):301–307. doi: 10.1016/S0014-5793(97)00723-0. 9271225

[41] Chen W, Ding R, Tang J, Li H, Chen C, Zhang Y, Zhang Q, Zhu X. Knocking out SST gene of BGC823 gastric cancer cell by CRISPR/Cas9 enhances migration, invasion and expression of SEMA5A and KLF2. Cancer Manag Res. 2020;12:1313–1321. 10.2147/CMAR.S236374. 32110105 PMC7040191

[42] Gatto F, Barbieri F, Arvigo M, Thellung S, Amarù J, Albertelli M, Ferone D, Florio T. Biological and biochemical basis of the differential efficacy of first and second generation somatostatin receptor ligands in neuroendocrine neoplasms. Int J Mol Sci. 2019 Aug 13. 20(16):3940. doi: 10.3390/ijms20163940. 31412614 PMC6720449

[43] Franzen J, Georgomanolis T, Selich A, Kuo C-C, Stöger R, Brant L, Mulabdić MS, Fernandez-Rebollo E, Grezella C, Ostrowska A, et al. DNA methylation changes during long-term in vitro cell culture are caused by epigenetic drift. Commun Biol. 2021 May 19. 4(1):598. doi: 10.1038/s42003-021-02116-y. 34011964 PMC8134454

[44] Ohnmacht AJ, Rajamani A, Avar G, Kutkaite G, Gonçalves E, Saur D, Menden MP. The pharmacoepigenomic landscape of cancer cell lines reveals the epigenetic component of drug sensitivity. Commun Biol. 2023 Aug 9. 6(1):825. doi: 10.1038/s42003-023-05198-y. 37558831 PMC10412573

[45] Leiszter K, Sipos F, Galamb O, Krenács T, Veres G, Wichmann B, Fűri I, Kalmár A, Patai ÁV, Tóth K, et al. Promoter hypermethylation-related reduced somatostatin production promotes uncontrolled cell proliferation in colorectal cancer. PLOS ONE. 2015;10(2):e0118332. doi: 10.1371/journal.pone.0118332. 25723531 PMC4344335

[46] Patai AV, Valcz G, Hollosi P, Kalmár A, Péterfia B, Patai Á, Wichmann B, Spisák S, Barták BK, Leiszter K, et al. Comprehensive DNA methylation analysis reveals a common ten-gene methylation signature in colorectal adenomas and carcinomas. PLOS ONE. 2015;10(8):e0133836. doi: 10.1371/journal.pone.0133836. 26291085 PMC4546193

[47] Ampofo E, Nalbach L, Menger MD, Laschke MW. Regulatory mechanisms of somatostatin expression. Int J Mol Sci. 2020 June 11. 21(11):4170. doi: 10.3390/ijms21114170. 32545257 PMC7312888

[48] Gailhouste L, Liew LC, Hatada I, Nakagama H, Ochiya T. Epigenetic reprogramming using 5-azacytidine promotes an anti-cancer response in pancreatic adenocarcinoma cells. Cell Death Dis. 2018 May 1. 9(5):468. doi: 10.1038/s41419-018-0487-z. 29700299 PMC5920091

[49] Mori Y, Cai K, Cheng Y, Wang S, Paun B, Hamilton JP, Jin Z, Sato F, Berki AT, Kan T, et al. A genome-wide search identifies epigenetic silencing of somatostatin, tachykinin-1, and 5 other genes in colon cancer. Gastroenterology. 2006 Sep. 131(3):797–808. doi: 10.1053/j.gastro.2006.06.006. 16952549

[50] Andersen FG, Jensen J, Heller RS, Petersen HV, Larsson L-I, Madsen OD, Serup P. Pax6 and Pdx1 form a functional complex on the rat somatostatin gene upstream enhancer. FEBS Lett. 1999 Feb 26. 445(2–3):315–320. doi: 10.1016/S0014-5793(99)00144-1. 10094480

[51] Svendsen B, Holst JJ. Paracrine regulation of somatostatin secretion by insulin and glucagon in mouse pancreatic islets. Diabetologia. 2021 Jan. 64(1):142–151. doi: 10.1007/s00125-020-05288-0. 33043402

[52] Benuck M, Marks N. Differences in the degradation of hypothalamic releasing factors by rat and human serum. Life Sci. 1976 Oct 15. 19(8):1271–1276. doi: 10.1016/0024-3205(76)90263-0. 825697

[53] Gomes-Porras M, Cardenas-Salas J, Alvarez-Escola C. Somatostatin analogs in clinical practice: a review. Int J Mol Sci. 2020 Feb 29. 21(5):1682. doi: 10.3390/ijms21051682. 32121432 PMC7084228

[54] Gunther T, Tulipano G, Dournaud P, Bousquet C, Csaba Z, Kreienkamp H-J, Lupp A, Korbonits M, Castaño JP, Wester H-J, et al. International Union of basic and clinical pharmacology. CV. Somatostatin receptors: structure, function, ligands, and New nomenclature. Pharmacol Rev. 2018 Oct. 70(4):763–835. doi: 10.1124/pr.117.015388. 30232095 PMC6148080

[55] Clark OH, Ab B, 3rd, Jd B, et al. NCCN clinical practice guidelines in oncology: neuroendocrine tumors. J Nat Comp Cancer Network: JNCCN. 2009 Jul. 7(7):712–747. 10.6004/jnccn.2009.0050 19635226

[56] Salvi M, Borgo C, Pinna LA, Ruzzene M. Targeting CK2 in cancer: a valuable strategy or a waste of time? Cell death discovery. Cell Death Discovery. 2021 Oct 29. 7(1):325. doi: 10.1038/s41420-021-00717-4. 34716311 PMC8555718

[57] Chon HJ, Bae KJ, Lee Y, Kim J. The casein kinase 2 inhibitor, CX-4945, as an anti-cancer drug in treatment of human hematological malignancies. Front Pharmacol. 2015;6:70. doi: 10.3389/fphar.2015.00070. 25873900 PMC4379896

[58] Faust M, Schuster N, Montenarh M. Specific binding of protein kinase CK2 catalytic subunits to tubulin. FEBS Lett. 1999 Nov 26. 462(1–2):51–56. doi: 10.1016/S0014-5793(99)01492-1. 10580090

[59] Klein S, Meng R, Montenarh M, Götz C. The phosphorylation of PDX-1 by protein kinase CK2 is crucial for its stability. Pharmaceuticals. 2016 Dec 28. 10(1):2. doi: 10.3390/ph10010002. 28036027 PMC5374406

[60] Schwind L, Nalbach L, Zimmer AD, Kostelnik KB, Menegatti J, Grässer F, Götz C, Montenarh M. Quinalizarin inhibits adipogenesis through down-regulation of transcription factors and microRNA modulation. Biochim Et Biophys Acta General Subjects. 2017 Dec. 1861(12):3272–3281. doi: 10.1016/j.bbagen.2017.09.018. 28964816

[61] Nalbach L, Roma LP, Schmitt BM, Becker V, Körbel C, Wrublewsky S, Pack M, Später T, Metzger W, Menger MM, et al. Improvement of islet transplantation by the fusion of islet cells with functional blood vessels. EMBO Mol Med. 2021 Jan 11. 13(1):e12616. doi: 10.15252/emmm.202012616. 33135383 PMC7799357

[62] Lyon J, Manning Fox JE, Spigelman AF, Kim R, Smith N, O’Gorman D, Kin T, Shapiro AMJ, Rajotte RV, MacDonald PE, et al. Research-focused isolation of human islets from donors with and without diabetes at the Alberta diabetes Institute IsletCore. Endocrinology. 2016 Feb. 157(2):560–569. doi: 10.1210/en.2015-1562. 26653569

